# Single-Domain Antibodies and Their Formatting to Combat Viral Infections

**DOI:** 10.3390/antib8010001

**Published:** 2018-12-20

**Authors:** Dorien De Vlieger, Marlies Ballegeer, Iebe Rossey, Bert Schepens, Xavier Saelens

**Affiliations:** 1 VIB Center for Medical Biotechnology, 9052 Ghent, Belgium; dorien.devlieger@vib-ugent.be (D.D.V.); marlies.ballegeer@vib-ugent.be (M.B.); Iebe.rossey@vib-ugent.be (I.R.); bert.schepens@vib-ugent.be (B.S.); 2 Department of Biochemistry and Microbiology, Ghent University, 9052 Ghent, Belgium; 3 Department of Biomedical Molecular Biology, Ghent University, 9052 Ghent, Belgium

**Keywords:** virus, nanobody, formatting, Fc-domain, half-life

## Abstract

Since their discovery in the 1990s, single-domain antibodies (VHHs), also known as Nanobodies^®^, have changed the landscape of affinity reagents. The outstanding solubility, stability, and specificity of VHHs, as well as their small size, ease of production and formatting flexibility favor VHHs over conventional antibody formats for many applications. The exceptional ease by which it is possible to fuse VHHs with different molecular modules has been particularly explored in the context of viral infections. In this review, we focus on VHH formats that have been developed to combat viruses including influenza viruses, human immunodeficiency virus-1 (HIV-1), and human respiratory syncytial virus (RSV). Such formats may significantly increase the affinity, half-life, breadth of protection of an antiviral VHH and reduce the risk of viral escape. In addition, VHHs can be equipped with effector functions, for example to guide components of the immune system with high precision to sites of viral infection.

## 1. Introduction

The discovery of heavy chain-only antibodies in the serum of camels, first reported by Hamers et al. in 1993, opened the way for a new tool box for diverse therapeutic applications [1]. Sera from camelids such as camels, dromedaries and llamas contain conventional antibodies (IgG1 isotype) and, surprisingly, also antibodies that lack the light chain component as well as the first constant domain of the heavy chain (CH1) (IgG2 and IgG3 isotype). The epitope-binding unit of these so-called heavy chain-only antibodies thus consists of a single variable domain, called a single-domain antibody (VHH) or Nanobody^®^. Despite their small size (~15 kDa) these single domain binding units can have exquisite affinities and antigen-binding specificities [2,3,4,5]. Similar to the variable domain of conventional antibodies, VHHs consist of four constant framework regions (FR1–4) separated by three hypervariable complementarity determining loops (CDR1, -2 and -3). A distinct feature of VHH FR2 is the presence of a hydrophilic surface exposed patch that likely evolved to compensate for the loss of light chain binding. In addition, the CDR3 loop of a VHH often folds back over the site that normally interacts with the variable light chain. Moreover, the CDR3 of VHHs is more variable in length and typically somewhat longer than the CDR3 of conventional antibodies [5]. To compensate for the higher flexibility and otherwise entropically unfavorable binding to the target antigen, the CDR3 loop often forms a disulfide bond with the CDR1, CDR2 or FR2 [6,7,8].

The small size, single domain build-up and the presence of hydrophilic amino acids in FR2 go together with a typically high solubility and physical stability of VHHs. As a result, these proteins can withstand relatively harsh formulations and environments, have excellent tissue penetration capacities, can be formatted in multiple ways and can be efficiently produced at low cost in microorganisms [9]. Not surprisingly, given these appealing properties, VHHs directed against a number of viruses including influenza viruses, human immunodeficiency virus-1 (HIV-1), and human respiratory syncytial virus (RSV) have been isolated from immune, naïve or synthetic VHH libraries. Immune libraries can be generated based on peripheral blood lymphocytes isolated from a camelid that has been immunized with complete virus or a viral antigen of interest in a prime-boost strategy [10]. However, VHHs with reasonable target specificity can also be isolated from naïve libraries that were generated from a camelid that was not immunized with the target viral antigen of interest. Synthetic VHH libraries do not require any experimental animal handling. Such libraries are built based on a well characterized VHH of which the conserved FRs are retained and amino acids in the CDRs are altered by saturating site specific mutagenesis [11,12,13,14]. Immune libraries are often the first choice to isolate high affinity VHHs because natural somatic antibody maturation can create an enormous diversity. Antigen-specific VHHs are then usually isolated by phage, yeast or ribosome display [14,15,16,17].

**Figure 1:**
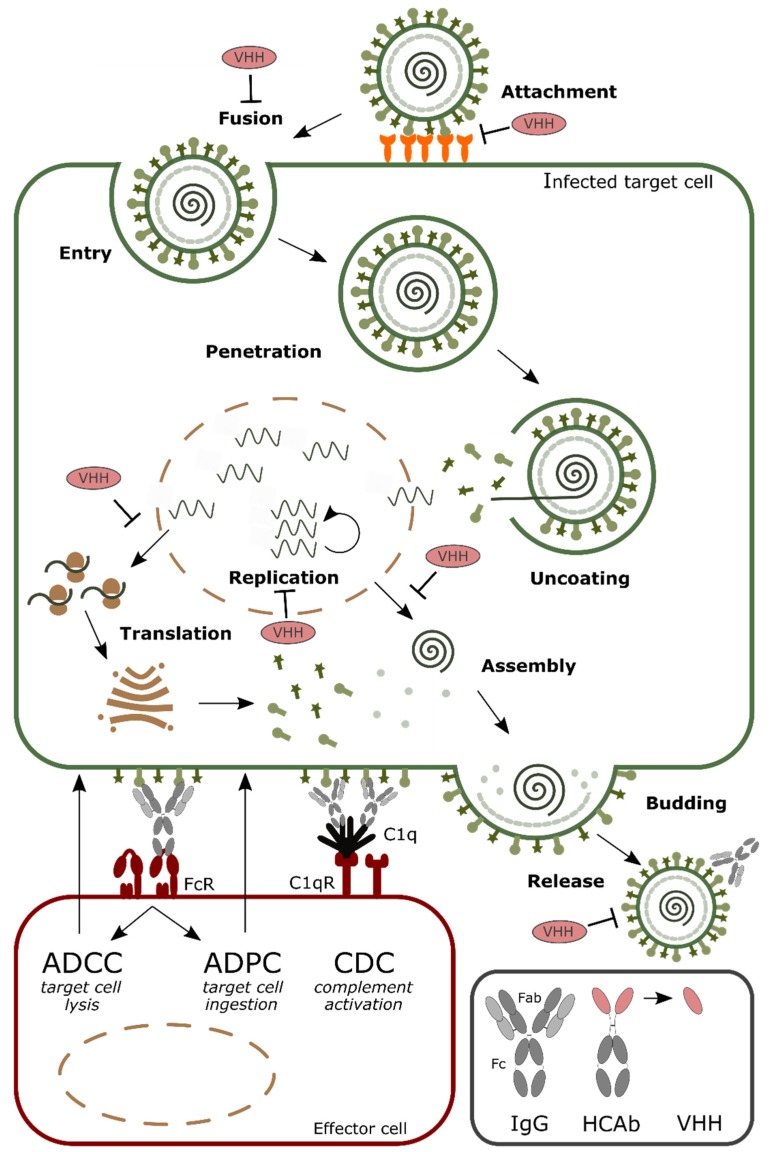
Schematic overview of the steps in a standard viral replication cycle that can be targeted by monovalent single-domain antibodies (VHHs) and antibody-mediated effector functions. General steps in viral replication include: (i) attachment and entry; (ii) penetration and uncoating; (iii) replication and translation of genomic viral RNA into proteins; (iv) assembly of virions; (v) budding and release. Antibodies (immunoglobulins or IgGs and heavy chain-only antibodies or HcAbs) employ different mechanisms to remove the infected target cells: (i) interact with Fc receptors (FcR) on effector cells to induce antibody-dependent cell-mediated cytotoxicity (ADCC) or antibody-dependent polymorphonuclear neutrophils (PMN)-mediated cytolysis (ADPC) and (ii) cell lysis through complement dependent cytotoxicity (CDC) by binding to the C1q receptor (C1qR).

Numerous virus-neutralizing VHHs have been described and different steps in the viral life cycle can be perturbed (Figure 1). For example, VHHs that prevent virus entry by blocking the receptor binding have been described for influenza (targeting the hemagglutinin (HA) protein), HIV (targeting gp120) and Middle East respiratory syndrome coronaviruses (MERS CoV) (targeting the spike proteins) [18,19,20,21]. Furthermore, a VHH that arrests the RSV fusion protein (F) in its prefusion state could prevent virus entry by inhibiting membrane fusion between virus and host cells [22]. When expressed within the target cell, the VHHs are often referred to as intrabodies, where they can affect, for example, viral replication and nuclear transport of viral ribonucleoproteins (vRNPs), as was shown for an anti-influenza nucleoprotein VHH, while a VHH against the HIV Rev protein could inhibit nuclear export of viral mRNA [23,24,25].

Apart from the direct antiviral activity that VHHs may have, VHHs can be formatted to serve as a targeting moiety to bring an effector function such as a toxin, an antiviral drug or an antibody-derived Fc domain to the site of a viral infection. VHH formatting can also be used to increase their half-life, the affinity for their target or to deliver the VHH to a certain compartment. At last, VHH formatting can be explored to develop new diagnostic tools for infectious diseases. Here we focus on the multitude of formats that have been applied to VHHs that target human viruses (Table 1).

## 2. Formatting of VHHs to Increase the Half-Life in Circulation

A single VHH molecule has a molecular weight of approximately 15 kDa, which is ten times smaller than a conventional IgG antibody (size around 150 kDa). Injected monomeric, dimeric and even trimeric VHHs are thus rapidly cleared from circulation by free glomerular filtration in the kidney (molecular weight cut-off 66 kDa) [26]. Such rapid removal, within a couple of hours after injection compared to 2–3 weeks for a conventional antibody, most often limits the therapeutic efficacy of a VHH [27,28]. As a result, VHHs have to be administered by infusion or repeat injection, or they are even restricted to loco-regional treatment [29]. It is important to note that there is no strict correlation between the molecular weight of a molecule and its half-life. For example, by genetic fusion of a VHH to an Fc domain the capacity to interact with the neonatal Fc receptor or FcRn is restored, which increases the retention of the VHH even more than expected based on the size [30]. It is clear that, in most cases, the half-life of the VHHs needs to be extended in order to obtain a maximal therapeutic efficacy. Prolonging the half-life will not only maintain the therapeutic threshold for a longer time but will also reduce the frequency of drug administration which will significantly benefit the patient [31]. Four major strategies, as discussed below, can be used to improve the pharmacokinetics of VHHs. In general, the goal is to limit their renal elimination and to recover the already available circulating VHH molecules.

A first frequently employed method aims to increase the size and the hydrodynamic radius of the VHH to avoid glomerular filtration. A simple way to accomplish this is by coupling two or three homologous or heterologous VHHs via a specific linker. However, even bivalent constructs are still rapidly cleared. A more complex method is the oligomerization of VHHs using a platform technology that allows to display VHHs. Different formats have been described, including the so-called fenobodies by Fan et al [32]. This display platform is based on ferritin, a spherical iron storage protein, to which, for example, broadly reactive anti-influenza VHHs have been anchored [33]. By doing so, the resulting VHH-displaying fenobody had a target affinity that was 360 times higher than the monovalent counterpart as well as a 10-fold higher half-life [32].

VHHs can also be chemically modified in order to increase the molecular weight. Such a chemical modification, for example with a polyethylene glycol (PEG) group, may also protect the VHH against proteases. The covalent attachment of PEG, a procedure that is often referred to as PEGylation and is approved by the Food and Drug Administration, to therapeutic proteins has been widely used to prolong the half-life of biopharmaceuticals. Interestingly, PEGylation of VHHs that can neutralize the highly contagious foot and mouth disease virus (FMDV, a picornavirus) did not only increase the half-life in guinea pigs but also significantly increased its neutralizing capacity [34]. The addition of a PEG molecule with a molecular mass higher than 50 kDa might result in accumulation of the VHH of interest in tissues, thereby reducing target access [35]. Next to this, chemical modification of a biotherapeutic can also result in reduced bioactivity or affinity of the molecule of interest [36,37]. Furthermore, there are safety concerns associated with the use of PEGylated protein drugs because PEG cannot be metabolized by the human body, and PEGylated biopharmaceuticals that are taken up by cells lead to vacuolation [38]. Combined with the relatively high cost of PEGylated molecules, the above-mentioned cautionary notes on the use of PEGylation to extend a drug’s half-life have roused the interest of companies to explore safer and more economical alternatives. The use of recombinant PEG mimetics that can be genetically fused to the molecule of interest is one example of such an alternative (reviewed in [29,39]).

A second approach to try to avoid renal clearance of biopharmaceuticals is based on the interesting observation that negatively charged small proteins remain longer in circulation than neutral proteins. Most likely, this is due to repulsion between the negatively charged molecule and the negatively charged glomerular basement membrane in the kidney [40]. There are several ways to add negative charges to proteins including the addition of sialic acid polymers (polysialylation) or hydroxyethal starch (HESylation) and by fusion with the highly sialyated beta carboxyterminal peptide (CTP) amino acid-residue found in the human chorionic gonadotropin (hCG) hormone [39]. Unlike PEG, these naturally occurring polymers are biodegradable [41]. It would be of interest to modify VHHs with such negatively charged polymers and to assess the outcome in terms of half-life and in vivo efficacy.

Another strategy to extend the half-life of a VHH is by coupling the VHHs to long-lived serum proteins or to building blocks that target these long-lived proteins. The long serum half-life of serum albumin, for example, results from its ability to escape from catabolism after cellular uptake. Once in the acidic endosomal compartment albumin is able to bind to the FcRn, which recycles the bound albumin back to the pH neutral extracellular milieu. Human serum albumin has a terminal elimination half-life of 17–19 days in human and is a preferred target of choice for direct or indirect fusion of VHHs [42,43]. A study by Hoefman et al. reported a significant increase in half-life of different mono-, di- or trimeric VHH constructs by fusing them to an albumin binding VHH. Following a single intravenous bolus administration, these half-life extended constructs could still be detected in the serum after 10 days in mice and 35 days in monkeys while constructs fused to an irrelevant VHH were no longer detectable within one day after administration [44]. Terryn et al. also genetically fused homo-and hetero-bivalent VHHs that target the glycoprotein G of Rabies virus with an albumin-specific VHH in order to extend the half-life. The albumin-binding capacity of the construct resulted in an approximately 100-fold higher systemic exposure and improved the median survival time after Rabies virus challenge of treated mice by several weeks compared with the bivalent constructs [45]. In this respect it is important to mention that the half-life of albumin in rodents and other small mammals, is only around 1–2.5 days, which is markedly shorter than the several days to weeks observed for different immunoglobulins (Igs) [46]. An additional advantage of fusion or binding to albumin is the fact that it might help to target VHHs to specific sites in the body since it has been reported that albumin can accumulate in tumors and inflamed tissues [47].

The half-life of Igs ranges from one to almost four weeks depending on the subclass and the isotype. Therefore, IgG-based fusion constructs are also widely used to extend the half-life of therapeutic proteins including VHHs. For example, the so-called VHH2, a genetic fusion between a FMDV-neutralizing VHH and a VHH that targets the porcine IgG, was constructed by Harmsen et al. [30,34]. When binding to porcine IgG was possible, a 100-fold increase in half-life was observed. Prophylactic intramuscular treatment with VHH2 also reduced viral load and shedding. Unfortunately, the FMDV challenge-associated clinical signs and transmission of the disease could not be prevented with this molecule in the model that was used. Altogether, the data obtained by Harmsen et al. showed that prolonging the exposure time is imperative for immunotherapy efficacy but additional fine tuning of other parameters is also important [34].

A fourth approach of extending the VHH half-life is to fuse the VHH of interest to the Fc region or other Fc-binding moieties of an IgG molecule. The extended half-life of such a chimeric antibody construct was demonstrated by Raj et al., who C-terminally extended a VHH that can neutralize the MERS-CoV with the Fc part of a human IgG2. The protein, that resembles a heavy chain-only antibody, showed an enhanced MERS-CoV neutralizing capacity and protection that correlated with sustained high levels of the VHH-Fc in circulation [48]. In parallel, Zhao et al. showed stable binding of a MERS-CoV neutralizing VHH fused with a human IgG1 Fc to recombinant MERS-CoV S1 spike protein in serum collected 10 days post-injection. This is in contrast to the monovalent format of the MERS-CoV VHH where no binding could be detected by day 10 [20]. In both cases, the improved protection against MERS-CoV infection with the Fc fused constructs was attributed to the increase in size by dimerization and thus the extended half-life. The effect of binding to the FcRn was not reported in these studies.

## 3. Increasing Valency to Improve Potency

In some instances, the affinity of an antiviral VHH picked up by an initial selection step still needs to be improved in order to enhance and broaden its neutralizing capacity. In addition, increasing the affinity might be important to reduce viral escape. To this end, one could change the affinity between the VHH and its target antigen by in vitro affinity maturation in which a second-generation library is constructed by introducing random mutations in a selected part of the VHH scaffold, usually the CDRs. For example, the affinity of a parathyroid hormone (PTH)-derived peptide-specific VHH that was isolated from a phage display library that was generated from a naive llama, could be enhanced 30-fold by selecting VHH derivatives from a library of VHHs with mutations that were randomly introduced in the CDR2 and CDR3 codons [49]. Another way to increase affinity is to introduce an avidity effect by joining two or more VHHs into multivalent constructs using flexible linkers. Several studies have demonstrated the beneficial outcome of that type of VHH formatting to improve affinity through avidity effects. In addition to increasing the binding avidity, formatting could also increase neutralizing activity by increased structural restriction when 2 sites on different protomers or proteins are linked by the VHH construct. Three types of multivalent VHH formats can be distinguished: monospecific fusions in which two identical VHHs recognizing the same epitope are joined, bispecific fusions in which two VHHs each recognizing a different epitope are connected, and formats where the VHH is fused to a protein moiety that has the tendency to dimerize or multimerize [50].

The strength of introducing avidity on the affinity of the VHHs for their target antigens was extensively examined by Hultberg et al. [21]. In this study, multivalent constructs were generated against the RSV F fusion protein, influenza H5N1 HA and Rabies G protein. Remarkably, linking two identical anti-F VHHs increased the RSV neutralizing capacity by approximately 4000. In addition, bivalent and trivalent anti-HA and bivalent anti- vesicular stomatitis virus (VSV) G VHHs had significantly higher neutralizing potency than their monovalent counterparts. Flexible Gly_4_Ser (GS) linkers ranging from 9 to 35 amino acid residues were tested. The optimal linker length needed to achieve an avidity effect most likely depends on the availability and spacing between the viral epitopes and needs to be empirically tested for each VHH combination. Trivalent anti-influenza HA VHHs with a 20 amino acid residue linker had lower neutralizing activity compared with constructs with a 10 amino acid residue linker. In contrast bivalent RSV F specific VHH with either a 10 or 20 amino acidlinker were equally potent in neutralizing RSV [21]. Further, Hulsik et al. developed a VHH (VHH 2H10) directed against the membrane proximal external region (MPER) epitope on the HIV gp41, the viral fusion protein [51]. Bivalent constructs in which two VHH 2H10 molecules were linked by a 15 or 17 GS linker, had a 20-fold higher binding efficacy compared with monomeric VHH 2H10. This increased affinity was associated with an increased breadth of neutralization: various HIV-1 strains that were resistant to monovalent VHH 2H10 could now be neutralized. In addition to using a GS linker, Cardoso et al. used the llama IgG2c hinge as a flexible linker to fuse two identical anti-influenza neuraminidase (NA) (H5N1) VHHs [52]. Similar to the above-mentioned studies, the antiviral potency of the bivalent format was significantly enhanced. Another example of a VHH that acquired increased virus neutralizing breadth by increasing its avidity, was reported for influenza A viruses as a target. A bivalent version of the VHH R1a-B6, generated by fusing two identical VHHs directed against the stem of HA of the 2009 H1N1 pandemic virus using a 30 amino acid GS linker, had a similar neutralizing potency against H1N1 and H5N1 viruses as monovalent R1a-B6. However, neutralization against a more divergent H9N2 virus strain was increased and the bivalent format even gained the ability to neutralize a H2N2 strain [18]. The authors proposed that this increase in antiviral breadth is the result of a slower off rate which can rescue low affinity interactions. Similarly, a broader antiviral spectrum was observed for bivalent formats of VHHs directed against the CD4-binding site or CD4-binding-induced site of the HIV-1 surface envelope glycoprotein (gp120) while no significant improvement of the neutralizing potency of these multivalent proteins in terms of IC_50_ was observed compared to those of the parental monovalent VHHs [53]. Apart from the in vitro increase in antiviral potency, the impact of the avidity effect was also observed in in vivo experiments. Ibañez et al. showed that an H5N1-neutralizing VHH in a bivalent format protected mice against a lethal H5N1 virus challenge and was at least 60-fold more effective in doing so than the monovalent counterpart based on lung viral titers in a dose-response comparison experiment [54]. Similarly, the intranasal administration of a bivalent RSV F-specific VHH and a bivalent rabies G-specific VHH showed increased in vivo potency compared to the monovalent counterpart [55,45]. Furthermore, ALX-0171, a homotrimeric VHH with high binding affinity for RSV F and potent RSV neutralizing activity, is currently being evaluated in a phase IIb clinical trial for the treatment of disease caused by RSV infections in infants. Interestingly, in in vitro experiments, the trivalent VHH outperformed palivizumab, a monoclonal humanized IgG1 antibody directed against the same epitope of the RSV F protein and currently used as a prophylactic anti-RSV treatment [56]. This could possibly be explained by the enhanced accessibility of the epitope due to the small size of the VHH, a more flexible GS linker compared with the antibody hinge or the presence of a third F-binding module in ALX-0171. To explore the impact of the avidity effect on the emergence of escape mutants in vitro, Palomo et al. serially passaged the RSV long strain in the presence of the monovalent or trivalent VHH [57]. This revealed that it was much more difficult for the virus to escape from the trivalent compared to the monovalent VHH. In addition, the trivalent format was still able to neutralize the RSV escape mutants.

Another way to overcome viral escape and broaden the neutralizing activity is to use a bispecific format, comprised of two VHHs that each target a different viral epitope. Hultberg et al. demonstrated that cross-neutralization was significantly improved with both RSV- and Rabies-neutralizing bispecific formats compared to the corresponding monovalent and bivalent monospecific constructs [21]. The generation of a single molecule (a head-to-tail fusion with a GS linker) with bispecific target recognition was key for this improvement because mere mixing of the monovalent VHHs in an equimolar ratio hardly increased the potency compared with the individual monovalent VHHs. Interestingly, the position of the VHHs relative to each other in the bispecific fusion construct can influence the potency [17]. Such an influence was also observed for bispecific VHHs (JM2x5 or JM5x2) directed against the CD4-binding site or CD4-binding induced site of HIV-1 gp120. The bispecific construct with JM5 VHH at the N-terminal and JM2 VHH at the C-terminal position outperformed the construct with the VHHs in the opposite position [53].

Finally, it is also possible to increase affinity and neutralizing potency by fusing a VHH with a protein moiety that has a tendency to oligomerize. For example, Tillib et al. fused a trimerizing variant of the leucine zipper domain derived from the yeast transcription factor GCN4 to the C-terminus of an anti-influenza HA (H5N2) VHH [58]. The formatted VHHs adopted a trimeric conformation and were approximately 100 times more active compared with the unformatted VHHs. Likewise, Boruah et al. used another type of coiled-coil forming domain to generate multivalent VHHs [59]. It is even possible to generate so called combodies, constructs in which the VHH is fused with the coiled-coil domain derived from the human cartilage oligomeric matrix protein (COMP48), which results in a pentavalent VHH format. Compared with the monovalent format, the combodies were able to neutralize an 85-fold higher input of Rabies virus pseudotypes in vitro. Since the pentamerizing building block in combodies is of human origin, they have a reduced risk to become immunogenic upon administration in humans compared with the yeast leucine zipper domain. Another oligomerization approach is the development of the so-called fenobodies, as described before [32]. A very attractive way to generate bivalent VHHs is by fusion to the Fc domain of a conventional antibody (an IgG or IgA). For example, Cardoso et al. fused influenza H5N1 NA-specific VHHs to a mouse IgG2a-derived Fc domain [52]. The Fc formatted VHHs showed a 250-fold higher NA inhibition and approximately 50-fold higher in vitro antiviral activity compared to the monovalent VHHs. An important advantage of a VHH-Fc fusion construct is the introduction of Fc receptor-dependent effector functions such as complement activation and the possibility to engage effector functions such as antibody-dependent phagocytosis activity (discussed in the next paragraph).

## 4. Arming VHHs with Effector Functions

Next to direct virus neutralization, conventional antibodies can also employ Fc-mediated effector functions to control viral infections. These effector functions include antibody-dependent cell-mediated cytotoxicity (ADCC), antibody-dependent cell-mediated phagocytosis (ADCP) and complement-dependent cytotoxicity (CDC); mechanisms that can eliminate infected cells or virions [60]. The importance of these effector functions is increasingly recognized for antibody mediated protection against infections with e.g., influenza virus, RSV, Zika virus and HIV [61,62,63,64,65,66,67]. VHHs lack the Fc region and therefore cannot facilitate such effector functions as such. However, thanks to their single domain nature, VHHs can be readily equipped with various effector functions through molecular or biochemical engineering. As such VHHs can be linked to Fc regions but also to toxins, liposomes, and other ligand binding scaffolds.

Fc regions (CH2-CH3) of different human and mouse antibody subclasses have been genetically fused to VHHs that target different viruses. As outlined above, an Fc fusion greatly increases the serum half-life and enhances target antigen binding through an avidity effect (Table 1). For some antiviral VHH-Fcs the importance of the acquired Fc-associated effector functions has been investigated, e.g., in in vitro assays. VHH VUN400 targets the second extracellular loop of the CXCR4 chemokine receptor and can prevent entry and replication of an HIV-1 strain that uses CXCR4 as a co-receptor [68]. Fusing VUN400 to the CH2-CH3 domains of a human IgG1 antibody strongly increased the potency of this VHH to bind CXCR4 on the surface of T cells and to prevent HIV-1 infection. In addition, VUN400-Fc enabled ADCC of CXCR4-expressing target cells as evidenced by activation of FcγRIIIa, induction of natural killer (NK) cell degranulation and selective killing of target cells by NKs. VUN400-Fc could also mediate complement-dependent killing of cells that expressed high levels of CXCR4. Whether those acquired effector functions can also contribute to protection (both in vitro and in vivo) by VUN400-Fc remains to be determined.

The potential impact of VHH-Fc effector functions in vivo has been well illustrated for VHHs targeting the rotavirus VP6 protein and the influenza NA and HA proteins. The anti-rotavirus protein 1 (ARP1) is a VHH directed against the conserved rotavirus inner capsid protein VP6. Whereas conventional antibodies directed against the outer capsid protein VP7 can typically mediate serotype specific neutralization, antibodies directed against VP6 generally fail to do so. Remarkably, some non-neutralizing VP6 specific IgA monoclonal antibodies do protect mice against rotavirus infection when given systemically but not when delivered at the luminal side of the intestine [69]. When delivered intracellularly by protein lipofection, these antibodies were able to block rotavirus transcription by interfering with the rotavirus capsid structure [70]. As such non-neutralizing VP6-specific IgA could control rotavirus replication in vivo during transcytosis [69]. In sharp contrast to conventional VP6-specific antibodies, VHHs such as ARP1 that target VP6 can potently neutralize rotaviruses of diverse serotypes and control rotavirus infection in neonatal mice when fed orally [71,72]. A recently reported clinical trial demonstrated that therapeutic oral administration of yeast produced ARP1 was safe and effective in reducing diarrhea in male infants with severe rotavirus-associated diarrhea [73]. To try to further improve its protective capacity, the ARP1 VHH was fused to a mouse IgG1 Fc region and evaluated in vivo. Compared to monovalent ARP1 VHH, oral prophylactic treatment of Fc-ARP1 was far more effective in reducing the prevalence, duration and severity of diarrhea in rotavirus-infected neonatal mice pups. The observation that the (Fab’)_2_ fragment of Fc-ARP1 was much less effective illustrates that the capacity of Fc-ARP is not due to its bivalent nature, suggesting the importance of the Fc-associated effector functions. The role of Fc effector functions was further demonstrated by the reduced protective capacity of the Fc_N434D_-ARP1 variant. In contrast to Fc-ARP1, Fc_N434D_-ARP1 cannot interact with FcRn or the intracellular antibody receptor TRIM21 but is expected to have unaffected interaction with the Fcγ receptors. This suggests that next to direct neutralization, Fc-ARP may additionally mediate intracellular neutralization after internalization via FcRn and binding to TRIM21. In the cell, the intracellular antibody receptor TRIM21 can recognize virus–antibody complexes and target these for proteasomal degradation and initiate antiviral innate immune responses [74]. As the Fc_N434D_-ARP1 was still more potent than the ARP1 VHH, other Fc effector functions might also contribute to the protection provided by Fc-ARP1. 

The potential contribution of Fc effector functions in VHH-Fc mediated protection was also observed for a non-neutralizing Fc-VHH directed against the H5N1 NA protein [52]. We isolated H5N1 NA binding VHHs that can block the NA activity and consequently also in vitro viral replication. Bivalent formats of VHHs with NA inhibiting activity generated by head-to-tail or Fc fusion were at least 200-fold more potent than their monovalent counterparts at inhibiting NA activity and at least 30-fold in a plaque size reduction assay. In contrast, VHHN1-7-VHHm and its Fc fusion format bind H5N1 NA but fail to inhibit NA activity and viral replication in vitro. However, despite the lack of detectable anti-viral activity in vitro, prophylactic treatment of mice with N1-7-VHH in its Fc fusion format but not as a monovalent VHH could partially protect against an otherwise lethal H5N1 influenza virus infection. This suggests that the protective activity of pN1-7-VHH-Fc is dependent on its Fc effector functions.

More direct evidence for the importance of the Fc effector functions was recently reported for a multivalent VHH-Fc protein that can provide very broad protection against influenza A and B virus infection [75]. In this study, the experimental molecule MD3606 is comprised of a head-to-tail fusion of four different VHHs linked to a human IgG Fc region. Separately, these VHH can potently neutralize group 1 influenza A viruses, group 2 influenza A viruses, Victoria lineage influenza B or Yamagata lineage influenza B viruses. Prophylactic treatment of mice with MD3606 could protect them from an infection with diverse influenza A and B viruses at a challenge dose that was lethal for control treated animals. Leucine to alanine mutations in the Fc regions (LALA for human IgG1 and IgG2σ for mouse IgG2a MD3606 variants) that abrogate the interactions with Fcγ receptors and C1q (thereby eliminating ADCC, ADCP and CDC activity) substantially decreased the protective capacity of MD3606. This clearly demonstrates the importance of the Fc effector functions for the antiviral activity of VHH-Fc proteins.

Next to fusion with Fc regions also other approaches can be used to arm VHHs with Fc effector functions. For example, Sun et al. fused a conventional anti-CD4 antibody to a VHH that targets a HIV-1 gp120 co-receptor binding site that is exposed upon CD4 binding [77]. This antibody-VHH chimera was more efficient in blocking HIV-1 infections of T cells than the VHH as such. If the Fc region in this antibody-VHH construct could also engage effector functions was however not investigated. Gray et al. linked a HER-2-specific VHH to dinitrophenyl (DNP) which can recruit anti-DNP antibodies that are omnipresent in human sera. These DNP-fused VHHs could engage peripheral blood mononuclear cells (PBMCs) to kill HER-2 expressing target cells by ADCC [88]. Alternatively, a VHH that specifically targets the human FcγR III (CD16) fused to a tumor antigen-specific VHH could kill tumor cells by inducing ADCC and reduce tumor growth in mice that were xenografted with tumor cells and human PBMCs [89]. These last approaches might be of interest for arming virus-specific VHHs with various Fc effector functions.

Next to exploiting host Fc effector functions, VHHs can also be equipped with foreign effector functions. A VHH targeting the herpes simplex type 2 (HSV-2) glycoprotein D can bind to cells that express this protein but fails to neutralize HSV-2 infection in vitro. However once fused to the cytotoxic domain of *Pseudomonas aeruginosa* exotoxin A, this VHH (R33ExoA) can efficiently kill HSV-2 infected cells and as such reduce viral replication [78]. Fusion of CD7- and CD38-specific VHHs with toxins has been explored as a strategy to respectively control T-cell acute lymphoblastic leukemia and multiple myeloma [90,91]. VHH-based immunotoxins could represent a valuable approach for the control of latent viral infections such as those caused by HSV-2. Virus-specific VHHs could also be used to bring a cargo comprised of e.g., liposomes loaded with an antiviral drug selectively to infected cells. Shielding a toxic drug in a liposome may reduce systemic toxicity while increasing the efficacy of the delivered antiviral at the site of infection. Wang et al. conjugated the neutralizing HIV-1 gp120 specific VHH J3 to liposomes. Whereas non-covalent conjugation did not interfere with the neutralization by VHH J3, covalent conjugation of J3 to liposomes did. Although the reverse transcriptase inhibitor dapivirine encapsulated in liposomes had higher antiviral activity than free dapivirine, conjugation of J3 to dapivirine loaded liposomes did not further increase the antiviral activity [79]. Further optimization could potentially lead to a more pronounced antiviral therapy.

## 5. Targeting and Delivery of VHHs

In some cases, VHHs are only effective or needed in a certain organ, cell type or cell compartment. Multiple formatting options are available to specifically target or deliver VHHs to one of these regions, e.g., linkage to antibodies or peptides, provide posttranslational modifications or in situ expression by bacterial vectors.

Delivery of VHHs to an organ system such as the gastrointestinal tract can be easily achieved by the use of bacterial vectors. This approach was used to develop a therapy against rotavirus infections. Direct oral administration of the anti-rotavirus VHH ARP1 reduced rotavirus-induced diarrhea with 22.5% in a clinical study [71,73,92]. Delivering the VHHs with the use of a bacterial vehicle could result in enhanced and sustained protection. VHH expression by a bacterial vector can be accomplished by directing the VHH to the surface of the bacteria or by secretion of the VHH. *Lactobacillus paracasei* was engineered in two ways, resulting in either expression of mono- or bivalent ARP1 (or ARP3, another rotavirus-neutralizing VHH) on the bacterial surface, or the production of the monovalent ARP1 and ARP3 as secreted and/or surface displayed proteins [80,76]. The first strategy led to *Lactobacilli* which could reduce the rate of diarrhea in mice in both a prophylactic and therapeutic setting with a slightly improved efficacy for the bivalent VHH- compared to the monovalent VHH-expressing bacterium. Using the second, combined strategy, the virus could be captured by a VHH on the surface of the *Lactobacillus* whereas the secreted VHH could bind to a distinct epitope. The efficacy of this strategy has not yet been tested in vivo. Another group made use of the probiotic strain *Lactobacillus rhamnosus* GG, which was modified to display ARP1 on its surface [81]. Display of ARP1 on this *Lactobacillus* strain, which has intrinsic anti-rotavirus activity, resulted in ameliorated clinical parameters upon rotavirus challenge infection in mice compared to the original strain. None of these modified bacteria have been tested yet in clinical studies. Such studies are somewhat complicated from a regulatory perspective because it concerns the use of genetically modified micro-organisms. Also, the use of bacterial vectors is restricted to organs with a microbiome such as the gut, the skin, the nasal cavity and the vagina. Other organs can be targeted by linking the VHH of interest to a VHH that specifically binds to an organ-specific marker protein, which can enrich the VHH in the organ of choice upon systemic administration.

Next to targeting a complete organ, it can be advantageous to target only one cell type. This is the case with the anti-HIV iMabM36 antibody format, consisting of the HIV-neutralizing Ibalizumab (iMab) monoclonal antibody that was linked to two copies of the broadly HIV-neutralizing VHH m36 [77]. iMab is specific for CD4, the receptor of HIV. By linking the VHH to this antibody, the concentration of the broadly neutralizing VHH is enhanced at the site of infection resulting in a synergistic 10-fold antiviral effect compared to a mixture of the separate iMab and m36 VHHs.

At last, VHHs can also be targeted to a specific cell compartment to exert their function. Liu et al., for example, described a strategy to target HIV-neutralizing VHHs towards the lipid rafts on the cell membrane [82]. For entry and viral release, HIV relies on lipid rafts on the host cell membrane. Targeting anti-HIV VHHs to this site of infection could therefore increase the effectiveness of the VHHs. This is possible with the use of a glycosylphosphatidylinositol (GPI) signal, a hydrophobic moiety which guides proteins to the lipid rafts in the plasma membrane. A gene construct was made linking the coding information of the HIV CD4-receptor gp120-binding JM4 with the GPI signal sequence [53]. Transduction of CD4^+^ T cells with this construct resulted in broad and potent neutralization of HIV-1, while transduction with a construct coding for secreted JM4 did not neutralize any of the HIV-1 subtypes at all. A limitation of this technique is that the GPI-VHHs need to be produced by the CD4^+^ cells for them to be functional. This could be achieved by modifying patient-derived CD4^+^ cell with lentiviral vectors carrying the GPI-VHH sequence and then transfusing these cells back to the patient.

Another example of a GPI-linked VHH was described by Tiwari et al. who developed an anti-RSV strategy based on mRNA coding for an (GPI-linked) RSV-neutralizing VHH (F-VHH-4 described in Rossey et al.) [22,83]. In this strategy, in vitro produced VHH-coding mRNA is directly delivered to the lung resulting in a transient expression of the therapeutic VHH in the respiratory epithelial cells. By including a GPI anchor in the construct, the VHHs are directed to the epithelial cell surface, the site of RSV entry. Compared to a construct where the RSV-VHH is secreted, GPI-anchored RSV-neutralizing VHH could further reduce RSV infection in mice, even when administered seven days before infection. This is mainly due to the enhanced lung retention thanks to the GPI anchor.

Some viral targets may be located inside the infected cell and thus hard to reach by antiviral VHHs. This problem can however be overcome by linking the VHH to a cell-penetrating peptide such as penetratin. This approach has been applied for the development of anti-hepatitis C virus (HCV) VHHs. Several intracellular viral proteins were considered as targets: the viral RNA-dependent RNA polymerase, the NS3 helicase/NTPase and the multifunctional HCV NS4B protein [84,85,86,87]. In vitro, penetratin-linked VHHs directed against these targets could suppress HCV replication. These VHHs have not been tested yet in vivo. 

## 6. Antiviral Single Domain Antibodies as Tools for Diagnostic and Antigen Display

The sturdy nature, small size and ease of production make single domain antibodies very well suited as building blocks that can be used in applications such as diagnostics. However, in the context of virus-targeting VHHs, we came across very few examples in the literature that exploited VHH for the detection of viral antigens. One explanation for this paucity of studies is probably the tremendous sensitivity of nucleic acid-based detection methods for the diagnosis of a viral infection. However, in terms of speed, it is clear that rapid antibody-based detection methods outcompete DNA- and RNA-based detection methods. For some viruses that are known to manifest antigenic diversity in their structural proteins, a VHH-based detection method is a challenge unless a broadly reactive, yet highly specific VHH is available. Human noroviruses are an example of antigenically variable viruses, with more than 40 different genotypes that are classified into two main genogroups [93]. Furthermore, norovirus outbreaks can spread at an astonishing speed, warranting the availability of a rapid and robust diagnosis method, which could help to contain an outbreak as soon as possible after the first patients fall ill. One VHH that recognizes an epitope in the lower region of the protruding domains of the norovirus capsid that is conserved among genotype II noroviruses was developed into a tool for rapid diagnostics [94]. The norovirus capsid-binding VHH was modified with biotin and conjugated to gold, modifications that allowed its use in a rapid lateral flow immunoassay. Interestingly, compared with a commercially available ELISA for the analysis of human stool samples, the VHH lateral flow immunoassay had a higher specificity (86% compared with 67%), although one out of five cases were missed with the VHH set up (sensitivity of 80% for the lateral flow immunoassay compared with 100% for the commercial ELISA) [94]. The use of multiple VHHs, that ideally also can recognize genotype I noroviruses, would likely contribute to a higher sensitivity. Influenza viruses likewise display a high antigenic variability. Here also, a rapid detection method based on single domain antibodies would require those to be broadly reactive. A sandwich type ELISA has been proposed based on two single domain antibodies derived from a camel that had been immunized with inactivated semi-purified A/Texas/1/1977 H3N2 virus. These single domain antibodies were specific for the hemagglutinin of an H3N2 virus. In a sandwich ELISA, in which one of the single domain antibodies was tethered to magnetic beads and the second one was coupled with a reporter enzyme, the semi-purified H3N2 virus was detectable up to a minimal concentration of 50 ng/mL. It is unclear from this report if the single domain antibody pair was able to recognize multiple H3N2 strains [95].

A second VHH-based tool in the context of a virus has been developed by the group of David Rowlands. This group has a long standing in the design of hepatitis B core-based virus-like particles (VLPs) that can be expressed in robust expression systems such as those based on *E. coli* and plants. Recently, the group reported a so-called tandem fusion hepatitis B core VLP assembly method. When a single-domain antibody directed against green fluorescent proteins (GFP) was inserted in the major immunodominant region of the second hepatitis B core moiety of the tandem construct, recombinant VLPs were produced and purified that could capture GFP on their surface [96]. A similar VLP with a VHH directed against a nondisclosed virus was also generated by transient expression in *Nicotiana benthamiana* leaves. These so-called tandibodies could be used to display antigens for vaccination purposes. It will be interesting to see further reports on this technology for vaccine design purposes. Most likely, the hepatitis B core as well as the VHH will also be immunogenic. In addition, the VHH might shield an epitope on the displayed antigen.

## 7. Conclusions

Due to their unique properties such as high solubility, stability, ease of production and formatting flexibility, VHHs seem to be very well suited to develop high affinity reagents to fight human viral diseases. Currently eight VHHs are in clinical development (all from the company Ablynx), of which one, ALX-0171, a trivalent anti-RSV VHH currently tested in a phase IIb clinal trial, is directed against a viral target. Their single-domain build-up allows formatting in multiple ways to obtain “best-in-class” molecules. Head-to-tail fusion of identical VHHs or VHHs recognizing different epitopes or fusion to multimerizing protein moieties have successfully been demonstrated to enhance and broaden neutralization activity. The first strategy being exemplified by the superior antiviral activity and strain coverage of the trivalent VHH ALX-0171 compared with its monovalent counterpart. A key component in the generation of long-lasting antiviral therapeutics is the implementation of half-life extension techniques such as PEGylation, fusion to a serum albumin-binding VHH or fusion to an IgG Fc-domain. Clinical proof-of-concept of the extended half-life that is obtained in this way has been achieved for an anti-IL-6R and anti-TNF VHH fused to a serum albumin-binding VHH, used in the treatment of rheumatoid arthritis. PEGylated VHHs or VHHs fused to an Fc tail have not yet been tested in clinical trials. The fusion to an Fc tail however seems a promising approach since not only half-life is extended but also avidity and Fc effector functions are introduced. Moreover, several Fc fusion-based therapeutics are already on the market [97]. Alternatively, immune effector functions can also be added by fusion to an anti-CD3 VHH, resulting in the recruitment of T-cells. Next to improving affinity, extending the half-life and introducing effector functions, formatting can also improve VHH activity by targeting a certain organ, cell type or cell compartment. In this context, using a bacterial vector to deliver the VHH to specific organs seems an interesting approach to explore further. Finally, although VHHs are also well suited for the development of diagnostic tests, this is still a poorly explored area in the context of viral infections.

## Figures and Tables

**Table 1:** Different VHH formats and their functionality used to tackle various viral infections.

**VHH Format**	**Functionality**	**Virus**	**Reference**
Homobivalent VHHs 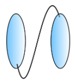	- Enhance and broaden antiviral activity	RSV Influenza Rabies HIV	Hultberg et al. [21] Schepens et al. [55] Detalle et al. [56] Palomo et al. [57] Hultberg et al. [21] Cardoso et al. [52] Hufton et al. [18] Ibanez et al. [54] Hultberg et al. [21] Terryn et al. [45] Hulsik et al. [51] Matz et al. [53]
Bispecific VHHs - VHH linked to anti albumin VHH 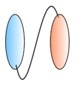 - VHH linked to anti IgG VHH 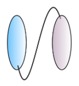 - VHH linked to VHH which binds different epitopes on same target 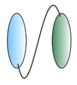	- Half-life extension - Half-life extension - Enhance and broaden antiviral activity	Rabies FMDV RSV Rabies HIV	Terryn et al. [45] Harmsen et al. [30] Hultberg et al. [21] Hultberg et al. [21] Matz et al. [53]
VHH Format	**Functionality**	**Virus**	**Reference**
PEGylation 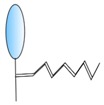	- Half-life extension	FMDV	Harmsen et al. [34]
VHH linked to IgG Fc region 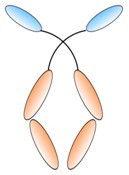	- Half-life extension - Enhance and broaden antiviral activity - Effector function	MERS Influenza HIV Rotavirus	Raj et al. [48] Zhao et al. [20] Cardoso et al. [52] Laursen et al. [75] Bobkov et al. [68] Günaydın et al. [76]
VHH linked to ferritin 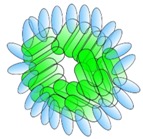	- Half-life extension	Influenza	Fan et al. [32]
VHH linked to GCN4 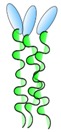	- Enhance and broaden antiviral activity	Influenza	Tillib et al. [58]
VHH linked to COMP48 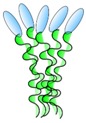	- Enhance and broaden antiviral activity	Rabies	Boruah et al. [59]
VHH linked to IgG 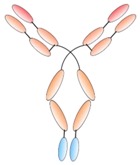	- Effector function - Targeting	HIV	Sun et al. [77]
VHH Format	**Functionality**	**Virus**	**Reference**
VHH linked to cytotoxic domain 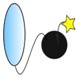	- Effector function	HSV-2	Geoghegan et al. [78]
VHH linked to liposome 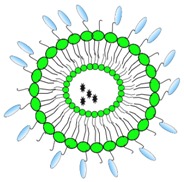	- Effector function - Targeting	HIV	Wang et al. [79]
VHH linked to bacteria 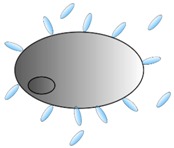	- Targeting	Rotavirus	Pant et al. [80] Günaydın et al. [76] Alvarez et al. [81]
VHH linked to GPI 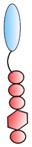	- Targeting	HIV RSV	Liu et al. [82] Tiwari et al. [83]
VHH linked to cell-penetrating peptide 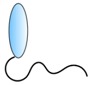	- Targeting	HCV	Thueng-in et al. [84] Phalaphol et al. [85] Glab-ampai et al. [86] Tarr et al. [87]

VHHs, single-domain antibodies; RSV: human respiratory syncytial virus; HIV: human immunodeficiency virus; IgG: immunoglobulin G; PEG: Polyethylene glycol; FMDV: foot-and mouth disease virus; MERS CoV: Middle East respiratory syndrome coronavirus; GCN4: amino acid starvation-responsive transcription factor; COMP48: human cartilage oligomeric matrix protein; HSV-2: Herpes Simplex Virus-2; GPI: glycosylphosphatidylinositol; HCV: hepatitis C virus.
